# Pregnancy-associated metabolic adaptations in circulating monocytes and macrophages favor clearance functions

**DOI:** 10.3389/fimmu.2026.1786324

**Published:** 2026-03-06

**Authors:** Fátima Merech, Daiana Rios, Ana Schafir, Ignacio Rojas Campión, Melisa Bentivegna, Juan Beauquis, María Catalina Lava, Eugenio Antonio Carrera Silva, Andrea Emilse Errasti, Melisa Fariz, Daniel Paparini, Aldo Squassi, Luciana D´Eramo, Rosanna Ramhorst, Claudia Pérez Leirós, Soledad Gori, Vanesa Hauk, Daiana Vota

**Affiliations:** 1 Universidad de Buenos Aires (UBA), Consejo Nacional de Investigaciones Científicas y Técnicas (CONICET), Instituto de Química Biológica de la Facultad de Ciencias Exactas y Naturales (IQUIBICEN-CONICET), Laboratory of Immunomodulation, Metabolism and Cell communication, Facultad de Ciencias Exactas y Naturales (FCEN-UBA), Buenos Aires, Argentina; 2 Universidad de Buenos Aires (UBA), Facultad de Ciencias Exactas y Naturales (FCEN-UBA), Departamento de Química Biológica, Buenos Aires, Argentina; 3 Universidad de Buenos Aires (UBA), Consejo Nacional de Investigaciones Científicas y Técnicas (CONICET), Instituto de Química Biológica de la Facultad de Ciencias Exactas y Naturales (IQUIBICEN-CONICET), Laboratory of Immunopharmacology, Facultad de Ciencias Exactas y Naturales (FCEN-UBA), Buenos Aires, Argentina; 4 Laboratory of Neurobiology of Aging, Instituto de Biología y Medicina Experimental (IBYME), Consejo Nacional de Investigaciones Científicas y Técnicas (CONICET) y Fundación IBYME, Buenos Aires, Argentina; 5 Instituto de Medicina Experimental (IMEX), Academia Nacional de Medicina, Consejo Nacional de Investigaciones Científicas y Técnicas (CONICET), Buenos Aires, Argentina; 6 Universidad de Buenos Aires (UBA), Instituto de Farmacología, Facultad de Medicina, Buenos Aires, Argentina; 7 Universidad de Buenos Aires (UBA), Facultad de Odontología, Cátedra de Odontología Preventiva y Comunitaria, Instituto de Investigaciones en Salud Pública, Buenos Aires, Argentina

**Keywords:** fatty acid oxidation, immunometabolism, lactate, macrophage, maternal immune homoestasis, monocyte, pregnancy

## Abstract

**Introduction:**

Pregnancy requires coordinated immunometabolic adaptations that allow maternal immune tolerance while preserving tissue remodeling and host defense. Circulating monocytes contribute critically to these processes, yet how gestation shapes their metabolic state and functional specialization remains incompletely defined.

**Methods:**

We investigated the metabolic and functional phenotype of maternal monocytes during earlymid pregnancy (1620 weeks of gestation) and explored the contribution of trophoblast-derived signals using an *in vitro* macrophage model and trophoblast-conditioned media.

**Results:**

Maternal circulation was enriched in CD14^+^CD16^+^ monocytes, accompanied by increased plasma lactate levels and elevated *ex vivo* lactate secretion by purified monocytes, without changes in mitochondrial mass or membrane potential. Monocytes from pregnant women displayed enhanced long-chain fatty acid uptake and increased expression of the fatty acid transporter CD36, while lipid droplet accumulation remained unchanged. Pregnancy-associated efferocytosis was dependent on fatty acid oxidation (FAO), as pharmacological FAO inhibition abrogated this response. Transcriptional profiling revealed differential regulation of TAM receptors, characterized by increased MERTK and reduced AXL expression, consistent with a homeostatic efferocytic program. Trophoblast-derived conditioned media recapitulated key features of this phenotype in macrophages, inducing fatty acid uptake, lipid dropletmitochondria colocalization, and upregulation of CPT1, DGAT1, LXRa and RARa. In this model, FAO was required to sustain ATP production and M2-like marker expression, while monocarboxylate transport was necessary for efficient efferocytosis and fatty acid uptake.

**Discussion:**

Together, these findings identify a coordinated immunometabolic program in maternal monocytes integrating glycolysis, lactate signaling, and FAO, likely instructed by trophoblast-derived cues, to enhance efferocytic and pro-resolving functions during pregnancy. This metabolic adaptation may represent a systemic mechanism supporting immune tolerance and tissue remodeling in early gestation.

## 1 Introduction

Pregnancy entails a unique immunological challenge: the maternal immune system must tolerate a semi-allogeneic fetus while maintaining the capacity to respond to pathogens and support tissue remodeling and repair. These changes emerge at implantation and intensify throughout gestation, integrating endocrine inputs, nutrient availability and placental-derived factors to sustain decidual remodeling, angiogenesis and fetal development (1, 2). Failure to maintain appropriate immune regulation at the maternal–fetal interface has been linked to the development of pregnancy complications such as preeclampsia (3, 4). Innate immune function is tightly constrained by metabolic state. Foundational work in immunometabolism has established that rewiring of central carbon metabolism shapes immune cell phenotypes: glycolytic dependency supports pro-inflammatory cytokine production and inflammasome activation, whereas oxidative phosphorylation and fatty acid oxidation (FAO) enable regulatory, reparative and pro-resolving programs (5–9). Although glycolysis is often linked to inflammatory activation and FAO to regulatory phenotypes, these associations are not absolute; metabolic pathway engagement is highly context-dependent and shaped by the specific stimuli and microenvironment encountered by immune cells (5). These principles are highly relevant in normal pregnancy since it is a state characterized by systemic metabolic adaptations, including progressive changes in lipid handling and insulin sensitivity that alter circulating nutrient availability and thus the metabolic milieu encountered by blood leukocytes (10–12). Monocytes are highly plastic circulating myeloid cells that differentiate into macrophages after extravasation into tissue. They are key effector cells not only in the initiation of an inflammatory response against an insult, but also in driving tissue repair, remodeling, and the maintenance of homeostasis. Maternal circulating monocytes and macrophages at the maternal–fetal interface contribute to apoptotic cell clearance, trophoblast invasion, and vascular remodeling functions, requiring tight metabolic control (11, 13). Recent reviews summarize how monocyte and macrophage distribution and function are remodeled during healthy and complicated pregnancies, highlighting both longitudinal shifts in subset frequencies and context-specific functional reprogramming (13–15).

In our earlier work, we characterized the immunometabolic profile of freshly isolated monocytes from pregnant women (16–20 weeks) and compared them with nonpregnant controls (16). We found that pregnancy monocytes exhibited increased glucose dependency and enhanced efferocytic capacity, together with elevated IL-10 production in the absence of changes in IL-1β levels. *In vitro*, several features of this pregnancy-associated phenotype, including the lack of metabolic reprogramming in response to LPS and the induction of IL-10 production in macrophages, were partially recapitulated by trophoblast-derived soluble factors, supporting the hypothesis that trophoblast signals shape the distinct metabolic and functional state observed ex vivo in maternal monocytes during early-mid gestation (16).

Building on these observations and on current models of immunometabolic regulation in mononuclear phagocytes, we aimed to understand how early-mid gestation shapes the metabolic and functional state of circulating monocytes, and whether placental signals are sufficient to imprint key features of this phenotype. To address this, we performed an integrated characterization of monocytes from pregnant women at 16–20 weeks and complemented these analyses with an *in vitro* macrophage model exposed to trophoblast-derived soluble factors.

Our results reveal an enrichment of CD14^+^CD16^+^ monocytes in maternal circulation at 16–20 weeks, increased fatty acid uptake and lactate secretion without changes in mitochondrial mass or membrane potential, and enhanced efferocytosis that depends on FAO. *In vitro*, trophoblast-conditioned medium aligns with many of the metabolic adaptations (increased fatty acid uptake, lipid droplet accumulation and upregulation of CPT1, DGAT1 and RARα) and rendered macrophages more reliant on FAO for ATP maintenance and anti-inflammatory marker expression. Collectively, these data support the existence of an early systemic immunometabolic adaptation in pregnancy, potentially mediated by trophoblast signals and involving retinoid and lipid pathways, that may promote tissue remodeling and immune tolerance during gestation.

## 2 Material and methods

### 2.1 Patients

A total of 40 pregnant women aged 20–35 years, between 16 and 20 weeks of gestation and under regular obstetric follow-up, were enrolled in the study together with age-matched nonpregnant women selected using similar social and epidemiological criteria. Pregnant participants were excluded if they had a diagnosis of hypertension; a pre-pregnancy body mass index (BMI) <18.5 or >30 kg/m² according to WHO criteria; diabetes mellitus; positive serology for syphilis, hepatitis B, Chagas disease, toxoplasmosis or HIV; or detectable antiphospholipid antibodies. Peripheral blood samples were collected, and ex vivo metabolic and functional analyses were performed on circulating monocytes in accordance with protocols approved by the Ethics Committee of the School of Dentistry (approval no. 20211157) and the Argentine Society of Clinical Investigation (SAIC approval no. 10469/25). Written informed consent was obtained from all participants prior to inclusion.

### 2.2 Peripheral blood mononuclear cells purification and monocyte isolation

Peripheral blood mononuclear cells (PBMCs) were isolated from peripheral blood samples (10mL) of pregnant women or age matched nonpregnant fertile volunteers through Ficoll Paque PLUS (GE Healthcare Cat. No. 17-1440-03) and immediately subjected to *ex vivo* metabolic, phenotypic and functional assays.

### 2.3 Monocyte and macrophage differentiation for *in vitro* experiments

Peripheral blood mononuclear cells (PBMC) from healthy women volunteers at reproductive age were isolated by Ficoll-Paque PLUS density and monocytes were isolated by centrifugation on a discontinuous Percoll as previously described (17). The purity of the isolated cell population (>85%) was evaluated by flow cytometry analysis after CD14 labeling on a FACS Aria II cytometer (BD Biosciences, RRID: SCR_018091). Data analysis was performed using FlowJo software (RRID: SCR_008520). For differentiation into M0 macrophages, monocytes were cultured for 5 days in RPMI-1640 medium supplemented with 10% BFS and 50 ng/mL recombinant human M-CSF (Miltenyi Biotec, Cat. No. 130-093-963).

### 2.4 Trophoblast-derived cell conditioned media

The human first trimester cytotrophoblast-derived cell line HTR-8/SVneo (HTR-8) (RRID: CVCL_7162) obtained from The American Type Culture Collection (ATCC) were maintained in culture flasks at 37 °C, 5% CO_2_ in Dulbecco’s modified Eagle’s medium and Nutrient Mixture F12 (DMEM-F12) (Thermo Fisher Scientific, Cat. No. 12400024) supplemented with 10% heat-inactivated fetal bovine serum (FBS, Internegocios FRA), and 100 U·ml^− 1^ streptomycin-100 μg.ml^− 1^ penicillin solution (Thermo Fisher Scientific, Cat. No. 15140122) (18, 19). To obtain conditioned media (Tb-CM) the trophoblast cells were grown to subconfluence in polystyrene plates as described previously (18). Medium was replaced with RPMI-1640 supplemented with 2% FBS and after 18 hours of culture the conditioned media were collected, centrifuged to eliminate cell debris, and stored at -80 °C until they were used.

### 2.5 Lactate concentration

Plasma samples were obtained from peripheral blood of pregnant women (16-20w) and women of reproductive age by centrifugation. Lactate levels were measured using a colorimetric assay kit (Wiener Lab, Buenos Aires, Argentina), following an adapted version of the manufacturer’s protocol. For quantification of lactate secretion by monocytes, cells were plated and cultured for 18 hours in RPMI-1640 medium supplemented with 2% fetal bovine serum (FBS), supernatants were collected and centrifuged to remove dead cells and cellular debris before the colorimetric assay (20).

### 2.6 Long chain fatty acids uptake

For quantification of long-chain fatty acids (LCFAs) uptake, PBMCs from pregnant and non-pregnant women were incubated with the fluorescent probe BODIPY-FL C12 (Molecular Probes–Life Technologies, CA, USA) at a final concentration of 5 μM for 10 minutes at 37 °C. The probe was previously conjugated with 0.1% fatty acid–free bovine serum albumin (FAF-BSA) in serum-free RPMI-1640 medium for 30 minutes at 37 °C. Following incubation with the probe, cells were washed twice with 0.2% BSA in PBS and resuspended in 2% FBS in PBS solution for flow cytometric analysis. For quantification of LCFAs by monocyte-derived macrophages, after differentiation cells were cultured for 18 hours in RPMI-1640 2% FBS +/- monocarboxylate transporters inhibitors α-Cyano-4-hydroxycinnamic acid (4CHC) or 7ACC2, then incubated with the fluorescent probe BODIPY-FL C12 as for PBMCs described above. Data were acquired using a FACS Aria II (BD Biosciences, San Jose, CA, USA) and analyzed with FlowJo software (FlowJo, RRID: SCR_008520, http://www.flowjo.com) (16).

### 2.7 Lipid droplets accumulation

PBMCs were washed twice and incubated with 2 μM of the fluorescent probe BODIPY 493/503 (Thermo Fisher Scientific) in PBS for 15 minutes at 37 °C and 5% CO_2_. Cells were then washed with cold PBS and stained with a PE-Cy7–conjugated anti-CD14 antibody for 20 minutes at 4 °C to identify monocytes. Macrophages were processed under the same conditions. After staining, cells were washed with PBS, resuspended in 2% FBS in PBS solution, and analyzed by flow cytometry. Data were acquired in a FACS Aria II cytometer^®^ (BD Biosciences RRID: SCR_018091) and were analyzed using the FlowJo software (FlowJo, RRID: SCR_008520, http://www.flowjo.com) (16, 21).

### 2.8 Analysis of membrane potential and mitochondrial mass

PBMCs obtained from pregnant women and women of reproductive age were incubated with the fluorescent probes MitoTracker CMXRos (Thermo Fisher Scientific, Cat. No. M46752) or MitoSpy Green (BioLegend Inc. Cat. No. 424805) to assess mitochondrial membrane potential and mitochondrial mass, respectively, at a final concentration of 250 nM in serum-free RPMI-1640 medium for 20 minutes at 37 °C and 5% CO_2_. Cells were then washed with PBS and stained with a PE-Cy7–conjugated anti-CD14 antibody for 20 minutes at 4 °C. Cells were washed, harvested, and re-suspended in 2% FBS in PBS to flow cytometry analysis. Membrane mitochondrial potential was normalized to mitochondrial mass (16, 22).

### 2.9 Colocalization of lipid droplets and mitochondria by confocal fluorescence microscopy

Quantification of BODIPY 493/503 (lipid droplets) and MitoTracker (mitochondria) immunofluorescence was performed using ImageJ software. Individual cell regions of interest (ROIs) were manually outlined on spatially calibrated images and background-subtracted prior to thresholding. Thresholds were applied to identify immunopositive areas for each fluorophore. Colocalization analysis between both markers was conducted using the JACoP plugin and expressed as Manders’ colocalization coefficients: M1, representing the fraction of BODIPY 493/503–positive signal overlapping with MitoTracker, and M2, representing the fraction of MitoTracker–positive signal overlapping with BODIPY 493/503 (23).

### 2.10 Efferocytosis

For pregnant women and women of reproductive age, 5x10^6^ PBMCs were plated for 1 hour in RPMI-1640 without FBS at 37 °C and after washing three times with PBS, cells were cultured for 18 hours in RPMI-1640 supplemented with 2% FBS in absence/presence of etomoxir 10 µM. Etomoxir was used at a low concentration selected to minimize reported off-target effects. For monocyte-derived macrophages, 4x10^5^ cells were differentiated and incubated for 18 hours in RPMI-1640 2% FBS in absence/presence of 1mM 4CHC or 10 µM 7ACC2 lactate transporters inhibitors. Medium was removed and RPMI-1640 10% FBS with latex beads, carboxylate-modified polystyrene, fluorescent yellow-green (Sigma, cat L4655) were added for 2 hours at 37 °C or at 4 °C as control. Media was removed, cells were washed with PBS and analyzed by flow cytometry as described earlier.

### 2.11 Macrophages phenotypic markers

Cells were stained with APC, PE, FITC, or PECy7-conjugated antibodies directed to surface markers CD14 (BD Biosciences Cat. No. 555399, RRID: AB_398596; BD Biosciences Cat. No. 347497, RRID: AB_400312; Thermo Fisher Scientific, Cat. No. 25-0149- 42, RRID: AB_1582276), CD86 (BioLegend Cat. No. 305406 (also 305405, 305438), RRID: AB_314526), CD209 (Thermo Fisher Scientific Cat. No. 17-2099-42, RRID: AB_11039758), and CD163 (Biolegend Cat No. 333605) CD206 (BD Cat No. 550889) CD36 (BD Cat. No. 555454). Cells were recovered, washed with a staining buffer, and incubated for 20 minutes at room temperature with antibodies in PBS supplemented with 2% FBS, then washed twice, and resuspended. Ten thousand events were acquired by flow cytometry and analyzed as described earlier (16, 20).

### 2.12 Intracellular ATP content

Monocyte-derived macrophages were cultured and treated ON with Tb-CM in the presence or absence of metabolic pathway inhibitors during the last 2 hours of stimulation (1mM 2-deoxy-D-glucose [2-DG], 1 µM oligomycin [O], or 10 µM etomoxir [Eto]). Culture medium was then removed, cells were washed and incubated for 5 minutes under gentle agitation with 50 μL of the ATP detection solution provided by a bioluminescence-based ATP assay kit (Promega, Madison, WI, USA) mixed with 50 μL of culture medium. Subsequently, 40 μL from each well were transferred in duplicate to a white 96-well plate optimized for bioluminescence detection. 40 μL were separated for protein precipitation with acetone and quantification by micro BCA protein assay kit (Thermo Fisher Scientific, Cat. No. 23235). Intracellular ATP content was measured using a Promega luminometer, according to the manufacturer’s instructions and then normalized to protein content.

### 2.13 Quantitative RT–PCR analysis

For monocytes from pregnant samples and non-pregnant controls, CD14+ monocytes were isolated from PBMCs of pregnant patients and healthy human volunteers using positive CD14 micromagnetic beads, MojoSort Human CD14+ Monocytes Isolation Kit (Biolegend, CA, USA), following manufacturer’s instructions and standardized protocol in our lab (24), RNA was isolated from at least 200.000 sorted CD14 monocytes using TriZol (Life Technologies, Carlsbad, CA, USA), and reverse transcription was performed to obtain copy DNA (cDNA) using 400 ng for monocytes of RNA in 20 µL of reaction volume, employing iScript cDNA synthesis kit (Bio-Rad, Hercules, CA, USA). Amplification was performed using 1 µL of cDNA in 10 µL of reaction volume, employing SsoAdvanced Universal SYBR Green mix and a CFX-Connect system (Bio-Rad, Hercules, CA, USA). The reaction was normalized to the expression level of the housekeeping gene *EEF1A1* and the specificity of the amplified products was checked through analysis of dissociation curves.

For monocyte-derived macrophages, total RNA was extracted using TriReagent (Molecular Research Centre, Ohio, US) according to the manufacturer’s instructions. 1 µg RNA was treated with DNAasa I following manufacturer’s instructions (Sigma-Aldrich, San Luis, MO, US) to avoid DNA contamination and the samples were reverse transcribed using a MMLV reverse transcriptase, RNAse inhibitor and oligodT kit (Promega Corporation, Madison, WI, US). cDNA was stored at −20 °C for batch analysis. Samples were incubated with SYBR Green PCR Master Mix (Roche, Basilea, Switzerland) and forward and reverse primers in Bio-Rad iQ5 Real-time PCR system. The expression was normalized to the endogenous β2-microglobulin gene control.

Relative gene expression was calculated using the 2^−ΔCT^ method. For in vitro experiments, fold changes were determined using the 2^−ΔCT^ method, with untreated cells. The primers used in this study are listed in **Table 1**.

**Table 1:** Primers used in qRT-PCR.

Gene	Forward (5’→3’)	Reverse (5’→3’)
*CPT1*	CTTGCCCTGAGACGGATTCT	AGCAGTGTTTCATCCCGAGC
*DGAT*	CGGTCCCCAATCACCTCATC	AGACTCGGAGTTCCACCAGT
*NR1H3* (LXRα)	ACCAGCTCCAGGTAGAGAGG	AACATCAGTCGGTCATGGGG
*RARA* (RARα)	GCCTGGACATCCTGATCCTG	CGTCTCCGCATCATCCATCT
*MER*	CTCTGGCGTAGAGCTATCACT	AGGCTGGGTTGGTGAAAACA
*TYRO3*	CGGTAGAAGGTGTGCCATTTT	CGATCTTCGTAGTTCCTCTCCAC
*AXL*	CCGTGGACCTACTCTGGCT	CCTTGGCGTTATGGGCTTC
*β2microglob*	AAGCAGCATCATGGAGGTTTG	GAGCTACCTGTGGAGCAACC
*Eef1A1*	TCGGGCAAGTCCACCACTAC	CCAAGACCCAGGCATACTTGA

### 2.14 Statical analysis

Data were analyzed using GraphPad Prism 6 software (GraphPad Prism (RRID: SCR_002798) San Diego, CA). Unpaired/paired Student’s t test, one-way or RM-one way ANOVA followed by Tukey’s or Dunnett’s multiple comparisons tests were used. When appropriate, Welch’s correction was applied. Data are presented as mean ± standard error (SE). Statistical significance was defined as p < 0.05.

## 3 Results

**Figure 1:**
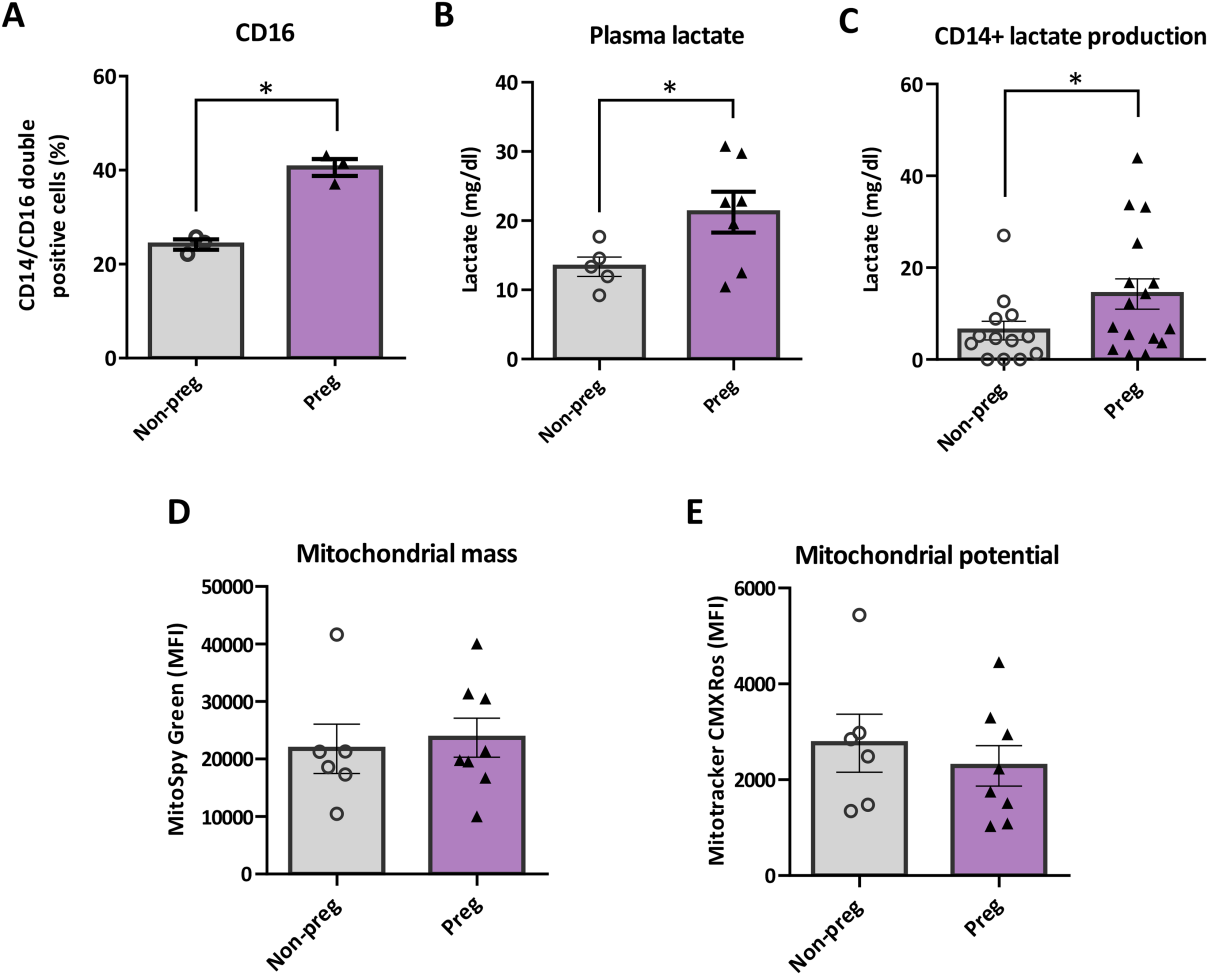
Monocytes from pregnant women display higher CD16 expression and lactate production. Plasma samples from pregnant women (16–20 w) or age-matched nonpregnant fertile women were obtained to evaluate lactate concentration. Peripheral blood mononuclear cells (PBMCs) were isolated as previously described. CD14 + cells were analyzed for CD16 expression and mitochondrial mass and potential or were plated for 18 hours to evaluate lactate production. Cells were incubated with CD14 and CD16 mouse anti-human antibodies **(A)**, Mitotracker CMXRos or Mitospy probes **(D, E)** and analyzed by flow cytometry. **(B, C)** Lactate concentration in plasma or supernatant were determined by colorimetric assay. Each point represents an individual sample of monocytes from Non-pregnant (white dots) or pregnant (black triangles) women. Results are expressed as Mean ± S.E.M. Paired t-test with or without Welch’s correction was performed, *p < 0.05.

CD16-expressing monocyte subsets (intermediate and non-classical) are functionally distinct from classical CD14^++^CD16^−^ monocytes: they show patrolling behavior, distinct cytokine profiles, and specialized phagocytic/efferocytic capacities in diverse contexts (25, 26). Given their metabolic features and tissue-remodeling roles, changes in CD16^+^ subset frequencies could meaningfully influence maternal tolerance and clearance functions during gestation; however, the timing and metabolic correlates of these subset shifts in early to mid-pregnancy remain incompletely defined. We detected a higher proportion of CD14^+^CD16^+^ non-classical cells, a subset reported in other contexts to display elevated glycolytic activity, in samples from pregnant woman at 16–20 w (**Figure 1A**). Consistently, we found higher plasma lactate concentrations in pregnant women, despite unchanged plasma glucose levels (**Figure 1B**). In relation to the increased glucose dependence previously observed in maternal monocytes at 16–20 w, an increased *ex vivo* lactate production was detected in supernatants from purified CD14+ monocytes from these samples (**Figure 1C**).

We previously reported that monocytes from pregnant women exhibit enhanced efferocytosis, which was inhibited by 2-deoxyglucose (a glycolysis inhibitor) but even more strongly by an inhibitor of oxidative phosphorylation. Considering that no differences were observed in mitochondrial mass or membrane potential compared with monocytes from nonpregnant women (**Figures 1D, E**), we asked whether long-chain fatty acid oxidation contributes to the acquisition of the maternal monocyte phenotype.

**Figure 2:**
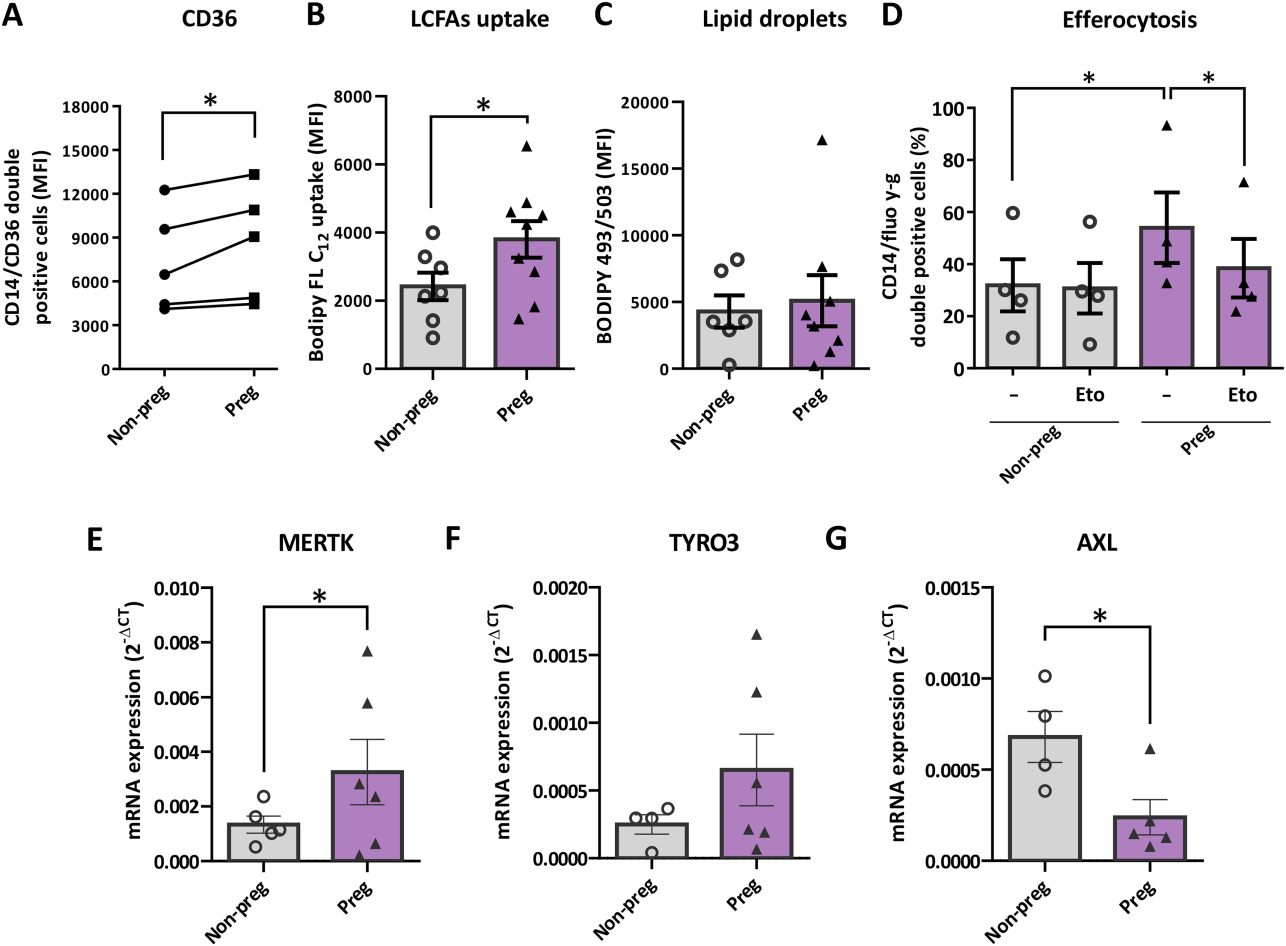
Monocytes from pregnant women present higher long chain fatty acids uptake and augmented efferocytic capacity dependent on fatty acid oxidation. PBMCs from 16–20 w or age-matched nonpregnant fertile women were obtained and immediately incubated with mouse anti-human CD14 and CD36 antibodies **(A)**, Bodipy FL-C_12_ **(B)**, Bodipy 493/503 **(C)** probes and analyzed by flow cytometry. **(D)** Cells were plated for 18 hours in absence/presence of FAO inhibitor Etomoxir (Eto), then efferocytosis was assessed by incubation with fluorescent latex beads and analyzed by flow cytometry. **(E–G)** CD14+ monocytes were isolated from PBMCs of 16–20 w or age-matched nonpregnant fertile women and phagocytic TAM (TYRO3, AXL and MERTK) receptors were analyzed by RT-qPCR. Each point represents an individual sample of monocytes from Non-pregnant (white dots) or pregnant (black triangles) women. Results are expressed as Mean ± S.E.M. Paired t-test or RM-one way ANOVA was performed, *p < 0.05.

We therefore evaluated the expression of the fatty acid transporter CD36, which in other models has been associated with the nonclassical phenotype, together with long-chain fatty acid uptake in monocytes from pregnant women. Both were significantly increased, without a rise in lipid droplet accumulation (**Figures 2A–C**). Moreover, when we evaluated efferocytosis in the presence of the FAO inhibitor etomoxir, we observed that FAO blockade also prevented the pregnancy-associated increase in efferocytosis (**Figure 2D**).

To further characterize the functional phenotype associated with pregnancy-induced metabolic adaptation, we next assessed the transcriptional expression of receptors directly involved in efferocytosis in isolated CD14+ monocytes. Transcriptional analysis of TAM receptors revealed a differential regulation of family members under our experimental conditions. MERTK expression was significantly increased, whereas AXL levels were reduced compared to control cells. In addition, TYRO3 showed a consistent trend toward increased expression, although this change did not reach statistical significance (**Figures 2E–G**).

These findings suggest that gestation promotes an expansion of the non-classical monocyte compartment (CD14+CD16+) that supports a tolerogenic, pro-resolving phenotype driven by a coordinated metabolic reprogramming in which glycolysis and fatty acid oxidation are simultaneously engaged.

**Figure 3:**
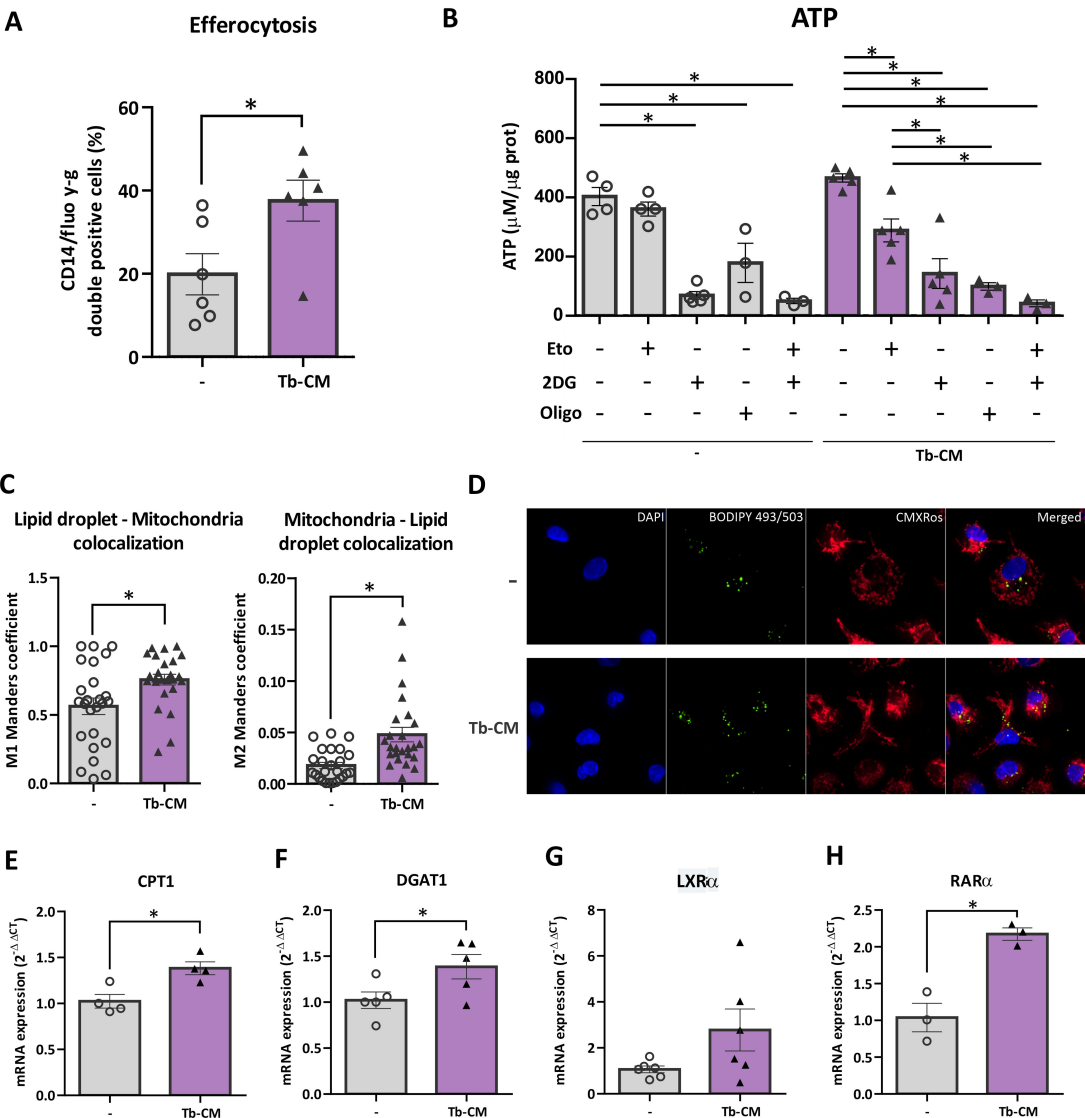
Trophoblast soluble factors induce macrophage metabolic reprogramming *in vitro* favoring fatty acid oxidation. Monocyte-derived macrophages (M0) were obtained and differentiated from healthy female donors as described in the Methods section. Cells were stimulated or not with trophoblast-conditioned media (Tb-CM) for 18 hours and were analyzed for efferocytic capacity **(A)**, intracellular levels of ATP upon inhibition of different metabolic pathways **(B)**; lipid droplet and mitochondria colocalization by confocal microscopy **(C, D)** and mRNA expression of **(E)** CPT1, **(F)** DGAT1, **(G)** LXRα, and **(H)** RARα. In **(C)**, Manders’ M1 coefficient represents the fraction of lipid droplet signal spatially associated with mitochondrial signal within the cell. Manders’ M2 coefficient represents the fraction of mitochondrial signal spatially associated with lipid droplet signal within the cell. Each point represents an individual sample of non-stimulated M0 macrophages (white dots) or Tb-CM stimulated macrophages (black triangles). Results are expressed as Mean ± S.E.M. One way ANOVA or t-test with/without Welch’s correction was performed, *p<0.05.

Given that in an *in vitro* model of maternal–fetal interaction we found that trophoblast-conditioned media from first trimester cytotrophoblast-derived cells (Tb-CM) induces long-chain fatty acids uptake in macrophages and enhances expression of M2-associated molecules (16), we next investigated the efferocytic capacity of macrophages and the contribution of major ATP-generating metabolic pathways in this model. We observed a significant increase in the efferocytic capacity of macrophages stimulated with trophoblast soluble factors (**Figure 3A**). By inhibiting specific metabolic pathways, we observed that intracellular ATP levels were differentially regulated: under basal conditions, FAO inhibition did not affect ATP generation, whereas glycolysis or global oxidative phosphorylation blockade markedly reduced ATP content. In contrast, in macrophages stimulated with Tb-CM, ATP production became sensitive to FAO inhibition, indicating activation of FAO under Tb-CM exposure (**Figure 3B**).

We also detected increased colocalization of cytoplasmic lipid droplets with mitochondria by confocal fluorescence microscopy (**Figures 3C, D**), along with elevated expression of the FAO enzyme CPT1, the lipid droplet-forming enzyme DGAT1, the nuclear receptor LXRα, and the intracellular retinoic acid receptor RARα, all associated with lipid metabolism and M2 polarization (**Figures 3E–H**).

**Figure 4:**
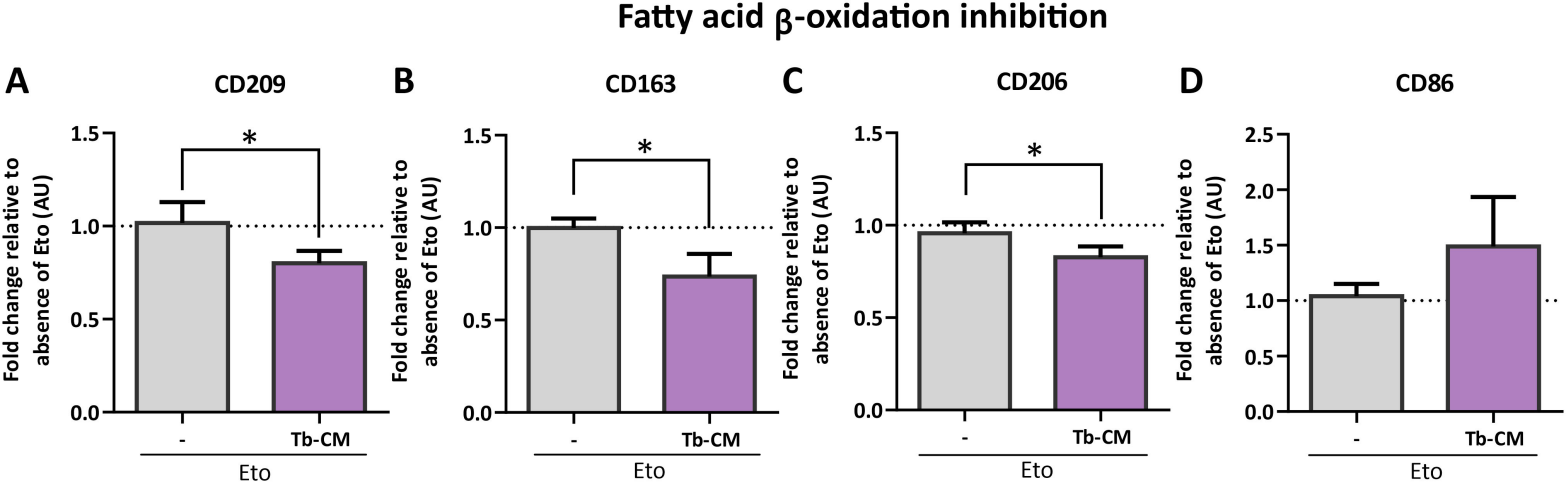
Fatty acid oxidation is required to sustain Tb-CM–induced expression of macrophage phenotypic markers. Monocyte-derived macrophages (M0) were obtained and differentiated from healthy female donors as described in the Methods section. Cells were stimulated or not with trophoblast-conditioned media (Tb-CM) in absence/presence of Etomoxir 10 µM (Eto) for 18 hours and then stained with mouse anti-human CD209 antibody (**A**, n=4), mouse anti-human CD163 (**B**, n=5), mouse anti-human CD206 (**C**, n=7), and mouse anti-human CD86 (**D**, n=8). Percentages of positive cells in the presence of Eto were normalized to the absence of the inhibitor. Results are expressed as Mean ± S.E.M. t-test was performed, *p<0.05.

We examined the direct link between lipid metabolic regulation and the acquisition of the Tb-CM-induced M2-like phenotype. In previous work, we showed that Tb-CM induces markers associated with a M2-like phenotype (16). Building on these findings, we next evaluated the impact of FAO inhibition on macrophage phenotypic marker expression by assessing fold changes in the presence of etomoxir. Notably, upon stimulation with Tb-CM, FAO blockade resulted in a marked reduction in the expression of M2-associated markers, including CD209, CD163 and CD206, to a significantly greater extent than that observed in unstimulated macrophages. In parallel, FAO inhibition in Tb-CM stimulated macrophages was associated with a trend toward increased expression of the pro-inflammatory marker CD86, an effect that was not evident in unstimulated cells (**Figure 4**). Together, these results indicate that FAO contributes to sustaining the Tb-CM induced phenotypic program, rather than for basal marker expression, highlighting a context-dependent role for lipid oxidation in shaping macrophage phenotype.

**Figure 5:**
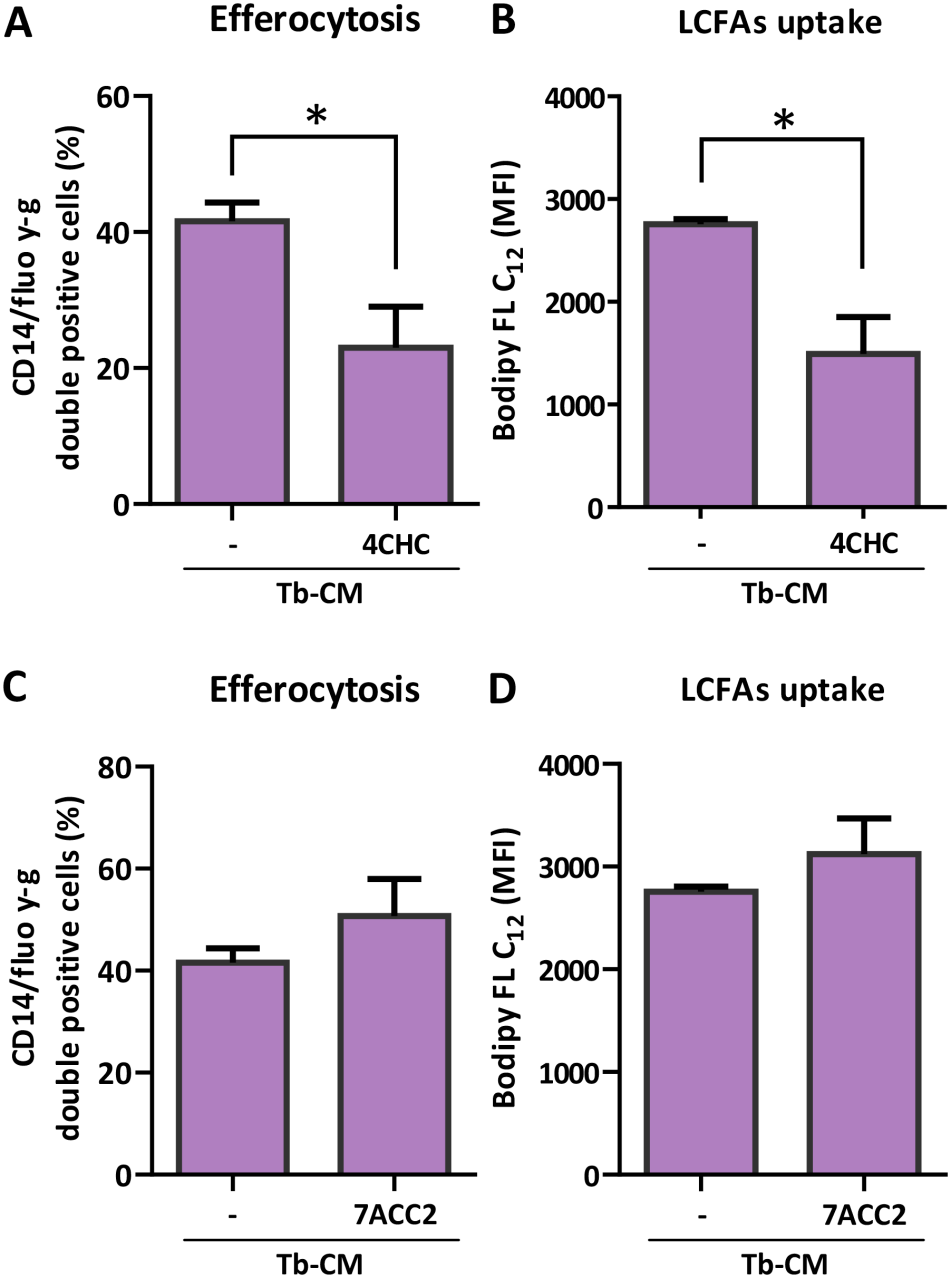
Lactate flux upon Tb-CM stimulation is required to induce efferocytosis and LCFAs uptake. Monocyte-derived macrophages (MO) were obtained and differentiated from healthy female donors as described in the Methods section. Cells were stimulated with trophoblast-conditioned media (Tb-CM) in absence/presence of monocarboxylate inhibitors 4CHC (1 mM) or 7ACC2 (10 µM), then efferocytic **(A, C)** capacity and LCFAs uptake **(B, D)** were evaluated as described previously. Results are expressed as Mean ± S.E.M of at least three independent assays. t-test was performed, *p<0.05.

Tb-CM was characterized by elevated lactate levels, as confirmed in our model (**Supplementary Figure 1**). To explore whether monocarboxylate-associated metabolic cues present in Tb-CM contribute to the modulation of macrophage metabolic and functional responses, pharmacological inhibition of monocarboxylate transport was performed, and macrophage efferocytic capacity was evaluated. Blockade of lactate flux using α-cyano-4-hydroxycinnamate (4-CHC) markedly abrogated Tb-CM-induced efferocytosis (**Figure 5A**), indicating that intact monocarboxylate transport is required to sustain these functional responses. In parallel, inhibition of monocarboxylate transport significantly decreased long-chain fatty acids uptake in Tb-CM stimulated macrophages (**Figure 5B**). In contrast, inhibition of lactate uptake using 7-AAC2 did not significantly affect efferocytosis or fatty acids uptake in Tb-CM–treated macrophages (**Figures 5C, D**). These results suggest that lactate handling, rather than lactate uptake *per se*, is critical for the acquisition of a pro-efferocytic M2-like phenotype. While global inhibition of monocarboxylate transport impairs both glycolytic flux and fatty acid metabolism, selective inhibition of lactate uptake preserving lactate export promotes metabolic rewiring toward fatty acid oxidation, tending to reinforce efferocytosis.

## 4 Discussion

Pregnancy requires a finely tuned immunological state in which maternal myeloid cells support tissue remodeling, trophoblast invasion and tolerance. Disruptions in these regulatory circuits contribute to complications such as preeclampsia and fetal growth restriction (3, 4). Within this context, our data provide mechanistic evidence that maternal circulating monocytes during early–mid gestation undergo coordinated metabolic adaptation—marked by increased glycolytic activity together with enhanced long-chain fatty-acid uptake and reliance on FAO—that expands their efferocytic and regulatory potential. These findings extend previous observations of elevated glucose dependence and enhanced efferocytosis in monocytes during pregnancy (16) placing lipid metabolism, and specifically FAO, as an additional metabolic axis regulating monocyte functional specialization.

The selective enrichment of CD14^+^CD16^+^ monocytes align with literature describing intermediate and non-classical subsets as specialized for patrolling behavior, efferocytosis and distinct metabolic programs, including high glycolytic activity (27). The observed increases in plasma lactate and *ex vivo* lactate secretion by maternal monocytes support a shift toward glycolytic metabolism, consistent with immunometabolic paradigms linking glycolysis to effector and certain pro-resolving immune functions (5–7, 28). These findings are in line with our earlier observation that pharmacological inhibition of glucose utilization compromises the efferocytic capacity of monocytes from pregnant women.

TAM receptor signaling, involving MERTK, TYRO3 and AXL, together with their ligands GAS6 and PROS1, plays a central role in immune homeostasis by negatively regulating innate immune responses, promoting apoptotic cell clearance, and facilitating the resolution of inflammation (29, 30). MERTK is recognized as the primary mediator of efferocytosis, whereas AXL expression is typically associated with inflammatory contexts and interferon signaling—a distinction that highlights the functional specialization within the TAM family (31). Under homeostatic conditions, MERTK serves as a constitutively expressed tolerogenic receptor that sustains efferocytosis (32, 33). Conversely, AXL expression is strongly induced by inflammatory stimuli, such as type I interferons and TLR3 ligands in macrophages (32) or in neoplastic inflammatory monocytes, as seen in Langerhans cell histiocytosis (34). Furthermore, MERTK and AXL exhibit a dichotomous or mutually exclusive expression pattern in specific macrophage populations and under different physiological or pathological conditions (32, 35, 36).

These observations align with our findings in CD14+ monocytes from pregnant women, where increased MERTK expression correlates with the downregulation of AXL. We hypothesize that the tolerogenic environment of pregnancy promotes MERTK and TYRO3 induction while suppressing AXL, thereby maintaining homeostatic efferocytosis in a non-inflammatory setting. Collectively, these data suggest that efferocytosis during pregnancy does not rely on damage- or inflammation-induced TAM signaling; rather, it is driven by tolerogenic pathways supported by MERTK/TYRO3 and metabolic rewiring. Importantly, efferocytosis is not merely a phagocytic function but drives metabolic reprogramming: while Morioka et al. stated that efferocytosis itself induces a solute carrier (SLC) program that promotes glucose uptake and lactate release in macrophages (37), recent work shows that effective and repeated efferocytosis requires metabolic flexibility, including activation of FAO and mitochondrial pathways to process lipid-rich cargo (38–40). Our finding that FAO inhibition abolishes the pregnancy-associated increase in efferocytosis underscores that maternal monocytes rely on FAO to carry out efficient clearance. Upregulation of CD36 and increased uptake of long-chain fatty acids further indicate engagement of lipid-utilizing metabolic pathways (8, 9, 41). The differential impact of pathway-specific metabolic inhibition on ATP production and efferocytic function under basal versus pregnancy-associated conditions supports an active redistribution of metabolic pathway usage rather than a static increase in metabolic capacity. This integrated view sets the stage for deeper mechanistic interrogation of the signaling pathways that couple metabolic rewiring to regulatory macrophage programs.

Using an *in vitro* trophoblast–macrophage interaction model, we show that trophoblast-derived soluble factors align several hallmarks of the *ex vivo* pregnancy-associated monocyte phenotype: enhanced LCFAs uptake, lipid droplet–mitochondria colocalization, and upregulation of CPT1, DGAT1 and RARα, key regulators of FAO, lipid handling and retinoid signaling. Notably, inhibition of FAO selectively disrupted the trophoblast-conditioned media-induced polarization toward an M2-like phenotype (CD209, CD163, CD206), while favoring expression of M1-associated markers such as CD86, indicating that FAO is instrumental for the acquisition of a trophoblast-driven regulatory macrophage phenotype (40, 42, 43). In line with this, as we previously reported, Tb-CM can bias monocytes toward an M2-like regulatory profile, supporting the concept that trophoblast-derived signals have the capacity to instruct myeloid cell differentiation.

Together, these observations suggest that trophoblast-derived signals engage interconnected metabolic pathways in maternal myeloid cells, prompting us to further dissect the relative contribution of lipid oxidation versus upstream metabolic cues in shaping macrophage function. Lactate transport via MCTs regulates intracellular redox balance and metabolic rewiring, thereby influencing macrophage fate and function. Pharmacological inhibition experiments revealed that disruption of monocarboxylate transport selectively impaired MC-induced fatty acid uptake and efferocytosis, whereas the inhibition of lactate uptake alone did not elicit comparable changes, indicating a differential contribution of these metabolic pathways (**Figure 5**). These results indicate that Tb-MC induces a coordinated immunometabolic program in monocytes/macrophages that is primarily instructed by lactate-associated signaling rather than by enhanced fatty acid oxidation *per se*. Notably, the functional consequences of monocarboxylate transporter inhibition appear to be context-dependent, as MCT activity has been shown to either constrain or promote inflammatory responses depending on the metabolic state and activation program of macrophages (44, 45). The elevated lactate content of Tb-CM, together with the sensitivity of both fatty acid uptake and efferocytosis to monocarboxylate transporter inhibition, supports a central role for lactate flux in shaping macrophage functional responses. The differential effects observed upon inhibition of monocarboxylate transport versus the lactate uptake only suggest a sequential organization of metabolic control in macrophages exposed to trophoblast-derived signals. Nevertheless, the differential effect of fatty acid oxidation inhibition on anti-inflammatory markers expression on Tb-CM stimulated vs. unstimulated macrophages indicates that FAO acts as a permissive pathway that stabilizes the regulatory phenotype downstream of lactate-dependent rewiring. Importantly, these results are consistent with our *ex vivo* observations in pregnant women, where circulating monocytes display a distinct immunometabolic profile associated with regulatory and homeostatic functions, suggesting that the CM-induced program recapitulates physiological adaptations occurring during gestation in maternal circulation.

Overall, these findings support a model in which early gestational cues, likely a combination of trophoblast-derived lipids, retinoids and other soluble factors, induce a dual metabolic program (glycolysis + FAO) in maternal monocytes that enhances their clearance capacity and pro-resolving potential, contributing to maternal-fetal immune tolerance. This concept aligns with accumulating evidence that FAO is fundamental for regulatory macrophage functions and resolution of inflammation (8, 9, 46).

Longitudinal studies across gestation and analyses in complicated pregnancies (preeclampsia, gestational diabetes, fetal growth restriction) are needed to define how perturbations in this immunometabolic axis contribute to maternal-fetal pathology.

## 5 Conclusion

Maternal circulating monocytes present a metabolic and functional configuration that relies on both glycolysis and FAO to support pro-resolving capacity; the trophoblast–FAO axis emerges as a novel immunometabolic checkpoint for tolerance maintenance during mid pregnancy.

## Data Availability

The original contributions presented in the study are included in the article/**Supplementary Material**. Further inquiries can be directed to the corresponding author/s.
