# Supplementary material for: Pregnancy-associated metabolic adaptations in circulating monocytes and macrophages favor clearance functions
Source: Front Immunol. 2026 Mar 6;17:1786324. doi: 10.3389/fimmu.2026.1786324 (PMC13002387; doi:10.3389/fimmu.2026.1786324)
Supplement: Supplementary file 1 [file DataSheet1.docx]

**Supplementary Material**

**Supplementary Figure 1:** Lactate concentration in trophoblast conditioned media. HTR8-Svneo cells were cultured for 18 h and lactate concentration in supernatant was measured as described in Methods section. Results are expressed as Mean ± S.E.M.


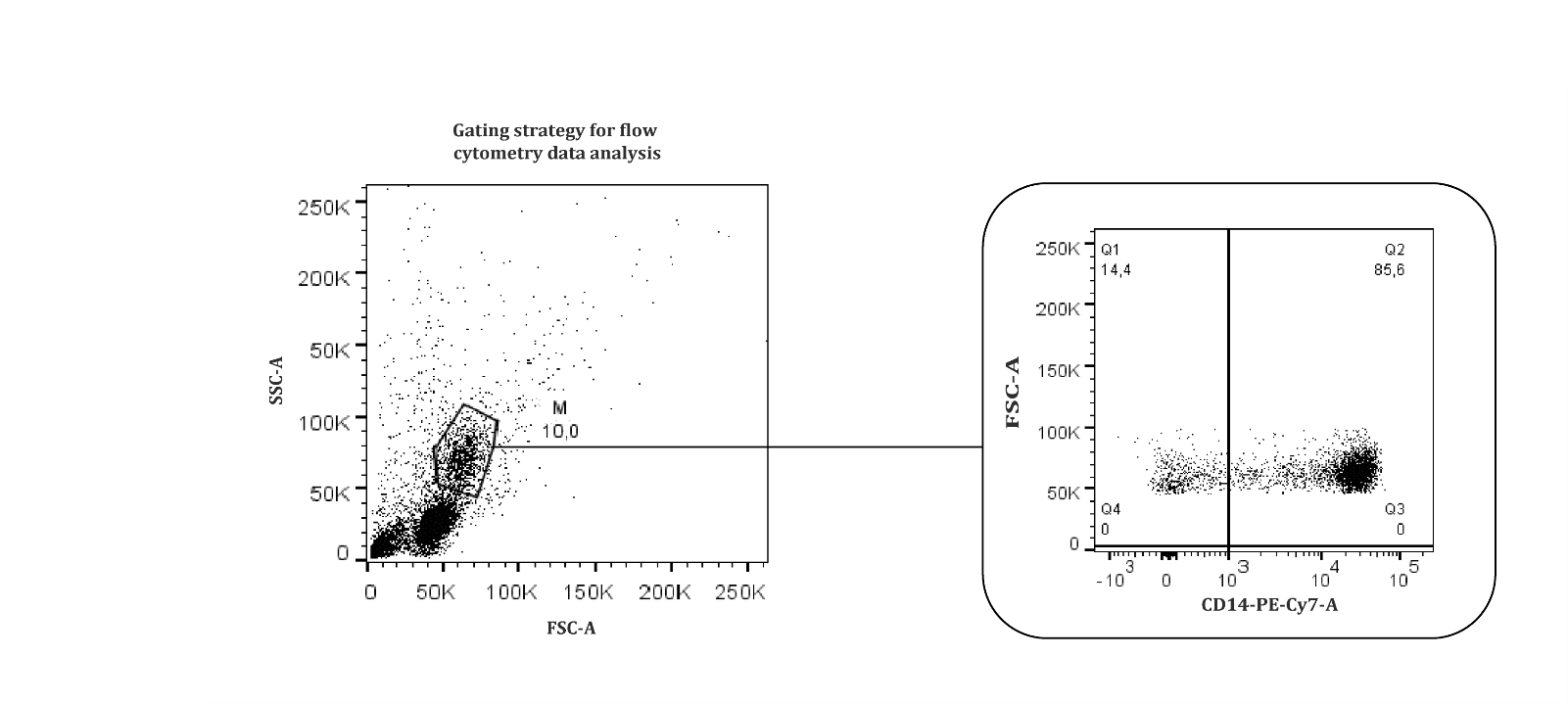


**Gating strategy** **for flow cytometry data analysis:** PBMCs were initially gated based on FSC-A vs SSC-A to define the leukocyte population of interest and exclude debris. CD14⁺ cells were then selected within this population for subsequent fluorescence analysis.
