# Supplementary material for: Pregnancy-associated metabolic adaptations in circulating monocytes and macrophages favor clearance functions
Source: Front Immunol. 2026 Mar 6;17:1786324. doi: 10.3389/fimmu.2026.1786324 (PMC13002387; doi:10.3389/fimmu.2026.1786324)
Supplement: Supplementary file 2 [file DataSheet2.pdf]

frontiers The International Society for the Advancement of Cytometry (ISAC) has highlighted the importance of including comprehensive methodological information to ensure data reproducibility and reliability. In line with this, Frontiers in Immunology now requires authors to submit a checklist for manuscripts that involve flow or mass cytometry. This checklist helps standardize the reporting process, improving the quality and transparency of published data. By doing so, we support scientific progress, making it easier for other researchers to replicate and validate experiments.

This form should be submitted with any manuscripts using flow or mass cytometry.

Sample/specimen/material description

☐ Total blood

X PBMCs

☐ Organ digests

Other: In vitro–differentiated human monocyte-derived macrophages

Did the samples suffer any treatment before or after incubation with the antibodies?

☐ Drug ______________________________________

☐ Cell permeabilization _______________________

X Dye: BODIPY-FL C12; BODIPY 493/503 ; MitoTracker CMXRos; MitoSpy Green; Fluorescent Latex Beads_________________________

☐ Propidium iodine

☐ Not applicable

Other: Trophoblast-derived cell conditioned media and/or Metabolic pathways inhibitors

Instrument and antibodies

Name of the Cytometer: FACS Aria II cytometer (BD Biosciences, RRID:SCR_018091)

Antibodies and targets	Fluorochrome/ Metal	Catalog number/Company
E.g. anti-CD4	FITC	Cat. XXX/ XXX ltd.
Anti-CD14	APC	Cat. No. 555399/ BD Biosciences

Anti-CD14	PE	Cat. No. 347497/BD Biosciences
Anti-CD14	PECy7	Cat. No. 25-0149-42/Thermo Fisher Scientific
Anti-CD86	PE	Cat. No. 305406, 305405, 305438/ BioLegend
Anti-CD209	APC	Cat. No. 17-2099-42/Thermo Fisher Scientific
Anti-CD163	PE	Cat. No. 333605/ BioLegend
Anti-CD206	APC	Cat. No. 550889/BD Biosciences
Anti-CD36	FITC	Cat. No. 555454/BD Biosciences
Anti-CD16	FITC	Cat. No. 302005/ BioLegend
BODIPY-FL C_{12}	FITC	Cat. No. D3822/Thermo Fisher Scientific
BODIPY 493/503	FITC	Cat. No. D3922/Thermo Fisher Scientific
MitoTracker CMXRos	PE	Cat. No. M46752/Thermo Fisher Scientific
MitoSpy Green	FITC	Cat. No. 424805/ BioLegend
Fluorescent Latex Beads	FITC	Cat. No. 1003532177/Sigma Aldrich

Data analyses

Name of the software: FlowJo software (FlowJo, RRID:SCR_008520, http://www.flowjo.com)

1. Reference gating strategy in the manuscript or supplementary material

The gating strategy has been added at the end of the Supplementary Material file.
